# Exploring the Social Determinants of Health and Health Disparities in Traumatic Brain Injury: A Scoping Review

**DOI:** 10.3390/brainsci13050707

**Published:** 2023-04-23

**Authors:** Leslie W. Johnson, Isabella Diaz

**Affiliations:** Department of Communication Sciences and Disorders, North Carolina Central University, Durham, NC 27707, USA; idiaz1@eagles.nccu.edu

**Keywords:** social determinant of health, health disparity, health inequity, traumatic brain injury, healthcare, outcome

## Abstract

Traumatic brain injury (TBI) is a global health concern, that can leave lasting physical, cognitive, and/or behavioral changes for many who sustain this type of injury. Because of the heterogeneity of this population, development of appropriate intervention tools can be difficult. Social determinants of health (SDoH) are factors that may impact TBI incidence, recovery, and outcome. The purpose of this study is to describe and analyze the existing literature regarding the prevailing SDoH and health disparities (HDs) associated with TBI in adults. A scoping review, guided by the Preferred Reporting Items for Systematic Reviews and Meta-Analyses (PRISMA) framework was used to explore three electronic databases—PubMed, Medline, and CINAHL. Searches identified peer-reviewed empirical literature addressing aspects of SDoH and HDs related to TBI. A total of 123 records were identified and reduced to 27 studies based on inclusion criteria. Results revealed race/ethnicity was the most commonly reported SDoH impacting TBI, followed by an individual’s insurance status. Health disparities were noted to occur across the continuum of TBI, including TBI risk, acute hospitalization, rehabilitation, and recovery. The most frequently reported HD was that Whites are more likely to be discharged to inpatient rehabilitation compared to racial/ethnic minorities. Health disparities associated with TBI are most commonly associated with the race/ethnicity SDoH, though insurance status and socioeconomic status commonly influence health inequities as well. The additional need for evidence related to the impact of other, lesser researched, SDoH is discussed, as well as clinical implications that can be used to target intervention for at-risk groups using an individual’s known SDoH.

## 1. Introduction

Traumatic brain injury (TBI) is a neurological disorder and a significant health concern impacting millions of individuals annually [1]. It is a leading cause of morbidity and mortality in the United States (US), and throughout the world [2,3,4]. Global incidence rates of those with a TBI have been estimated to reach up to 69 million people annually [1]. Injuries associated with TBI can range in severity from mild to severe, with most injuries being mild in nature. Globally, mild TBI represents roughly 55.9 million people each year, while severe TBI accounts for approximately 5.48 million people annually [1]. Long-term effects can render both physical and cognitive changes, and impact other aspects of daily living, such as relationships with friends and family, emotional processing, ability to live independently, and ability to maintain successful employment and community engagement. Roughly 5.3 million Americans today are living with impacts/impairments related to traumatic brain injury and this number is continuing to rise as the incidence of TBI escalates [5]. Numbers for those sustaining a TBI are projected to further increase, most likely due to growing elderly populations, as well as advances in medical care that often allow those with significant injuries better chances for survival. Many survivors live with significant disabilities, possibly contributing to socioeconomic burden, accounting for billions of dollars annually in both direct and indirect costs [5,6]. 

The TBI population is a heterogeneous group given the wide range of potential etiologies of TBI. Motor vehicles accidents, traumas, falls, sports-related injuries, and domestic violence are some of the more common etiologies of TBI. This wide range of mechanism of injury, as well as impairments that vary largely based on the location of damage within the brain, severity of initial injury, and the cascade of injuries that are likely to follow, make this population one of the more difficult to study. In the past several decades, investigators have looked at what factors may increase the risk for TBI, or impact intervention after TBI. Increasingly, research has demonstrated that social determinants of health (SDoH) play a significant role in TBI incidence, recovery, and outcome. 

### 1.1. Social Determinants of Health

The term SDoH is relatively new, only having developed within the past few decades; though, the influence of these factors on an individual’s ability to obtain quality healthcare has been prevalent long before the term was developed [7,8]. Social determinants are both modifiable and unmodifiable conditions related to an individual’s biology, culture, environment, role in society, and economic surroundings. Examples of unmodifiable SDoH are age and race/ethnicity. Modifiable SDoH are changeable, though the timeframe of change for many of these factors may be more long-term, rather than immediate or quick. They may occur over a life span, or between family generations. Examples of modifiable SDoH are, education level, health literacy ability, economic status, language spoken, and geographic location in which a person lives. A person’s SDoH can impact their overall health and well-being, and play a role in the potential health disparities, access to care, and treatment post injury that a person faces [9]. Despite the SDoH generally being related to non-medical factors, they have been noted to both positively and negatively influence the health outcomes [7,10,11]. In recent years, the amount of research on this topic has increased, specifically related to social factors [9,12]. An in-depth examination and clear understanding of an individual’s SDoH in conjunction with planning for health services and providing support to available resources is vital for better public health achievement, promoting access to care, and preventing health inequalities [11,12]. Chan and colleagues write that influences on health outcomes can impact a given individual or a population and are important factors that may impact health and healthcare-related services [11].

Social determinants of health include environmental conditions and conditions of daily living that can be influenced by society, social norms, policies, and/or politics, and have the power to shape what health and medical care looks like for individuals [10,13]. According to Braverman et al., SDoH can also be factors of health-related features of a neighborhood [10]. Being able to walk places, having access to transportation and recreation areas, accessibility to food and grocery stores with healthy/nutritious food options, and opportunities for physical activity all impact one’s overall health [14,15]. Place of birth, where one lives, safety of a neighborhood, what one does for a living, age, and accessibility to necessary daily items are all considered determinants of health that may affect quality of care [16]. Growing research has suggested that socioeconomics and financial wealth be included in aspects of an individual’s SDoH because of the important role these can play in determining the health outcomes [10,17]. Education level, income, employment status, air pollution, water quality, and overall wealth may seriously impact outcomes as well [9,18]. Language and literacy as a social determinant of health has also been studied. Research indicates that limited language and low literacy skills are generally associated with lower educational levels and can result in worse health outcomes [19,20,21]. Language barriers can also be associated with unequal health outcomes [22]. 

Consideration of how SDoH impact those with a TBI is important for both clinicians and researchers alike. Understanding that an individual’s modifiable and unmodifiable SDoH impacts their health and well-being is vital for those who work with people with TBI to know which groups may be more at risk for TBI, or likely to have poorer outcome after TBI. This is especially true for those with “unfavorable” SDoH, including low social economic status (SES), unemployment, and lower education, as these populations may have a greater prevalence of TBI [11]. Schillinger too believes SES and wealth play a role in SDoH stating that the determinants of health are “shaped by the current and historic distribution of money, power, and resources throughout local communities, nations, and the world” [23] (p. 22). Additionally, chronic stress associated with poverty, racism, or social isolation may lead to biological inflammation or other physiological changes that may increase the risk for TBI, or exacerbate symptoms related to TBI. Similarly, healthcare and rehabilitation services after TBI may vary for many patients depending on their geographic location, race/ethnicity, or insurance status. Braverman et al. notes that future research needs to better address and monitor the social factors impacting health in relation to policies, examine health effects of social factors across generations, and test multidimensional interventions [7]. Changes in these determinants, and in turn the resulting health disparities that may develop due to these, can be made. 

### 1.2. Health Disparities and TBI

The influence and interconnectedness of SDoH on health inequalities is important to note [23,24]. Social determinants of health are responsible for a variety of health inequities [23]. Policies addressing social determinants are important in trying to address health inequalities, poor health status of the parts of the population, and fluctuations in health [23]. A variety of terms are used when addressing this topic, including “health disparity”, “health inequity”, and “health care inequity”. Health disparities (HDs) are differences in health with regards to social or economic disadvantage and tend to negatively affect those who experience obstacles in these areas. Disparities are theoretically preventable and avoidable differences that tend to be more prevalent in marginalized groups in regards to distribution of health outcomes or the failing health of a population [7,23,25,26]. When comparing individuals of a given group to another, there are often differences in the burden of disease, injury risk, violence, disability, or opportunities for optimal health [23,25,26,27]. A difference in access to care, quality of care, and health coverage is also seen in groups with more detrimental SDoH [28]. Health disparities are often influenced by the SDoH of race/ethnicity, education, sex, sexual orientation, SES, insurance status, mental health, and place of residence [24,29,30]. The influence of SDoH on HDs and inequities has been noted in various populations, including heart disease, cancer, stroke, and TBI [11,31,32,33,34]. 

Multiple studies and articles have examined how HDs have influenced risk, mechanism of injury, acute hospitalization, assessment, rehabilitation, out-patient management, and long-term outcomes of those who have sustained a TBI [25,34,35,36,37]. Initial research has indicated HDs related to TBI are influenced by a variety of SDoH factors, including race/ethnicity, insurance status, geographical location, and SES. Racial/ethnic minorities have been noted to have longer wait times in the emergency department, higher discharge rate without being seen by a physician, and a lower likelihood of receiving follow-up care after discharge [34]. The cause of TBI varies among racial/ethnic groups as well with TBIs from acts of violence seen more often in Black and Hispanic individuals, whereas White individuals are more likely to have sustained a TBI from being hit by a car [38]. Insurance status is another SDoH contributing to potential HDs. For example, research has documented that patients with TBI who were insured have lower mortality rates than those uninsured [36,39,40]. Geographic location has been noted to influence TBI outcome by some researchers who note that unequal healthcare access may widen disparities given the scarcity of neurologists for post-TBI management in rural areas [41,42]. Finally, bias and stigma associated with various SDoH may also play a role in the treatment and care received post TBI [37]. Mukhtarzada and Monteith report that medical charts and records of non-Hispanic Black patients were more likely to contain stigmatizing language compared to non-Hispanic Whites [37].

### 1.3. Purpose of the Study

The purpose of this study is to (a) identify which SDoH are most commonly reported with TBI in adults, and (b) to categorize in detail the current research regarding HDs associated with TBI. With this information, the practicing clinician may be better able to discuss risk and prevention education related to TBI, and be able to better formulate appropriate intervention plans after TBI. The researcher may be able to better analyze the relationship between SDoH and HDs and bring attention to at-risk groups and policies that affect this population, thereby strengthening the programs dedicated to improving individual and community health. The scoping review methodology was selected given the variable and individualized nature of SDoH and the extensive reports of HDs within the TBI population. A scoping review allows researchers to collect information that is both specific and broad in nature, and is ideal for the clinical aspect of this study. The authors were particularly concerned with potential gaps in the current research related to SDoH; therefore, they chose the scoping review methodology and its data analysis framework to allow for tabulation of frequencies of reported SDoH and HDs. This helped the research team identify areas less studied, which may point to areas of potential clinical gaps. The scoping review technique has been established in the literature as a suitable means to study the heterogeneous TBI population, as demonstrated by the recent reviews focusing on cognitive function, social communication, mild TBI, and chronic cognitive impairment, and group-based delivery of intervention, among others [43,44,45,46]. 

## 2. Materials and Methods

### 2.1. Review Approach

Arksey and O’Malley demonstrated five essential stages of the scoping review methodology, including (1) identify the research question; (2) identify relevant studies; (3) study selection; (4) chart the data; and (5) collate, summarize, and report the results [47]. Others have since clarified and refined the process [48,49]. Additionally, the PRISMA (Preferred Reporting Items for Systematic Reviews and Meta-Analyses) Extension for Scoping Reviews checklist served as a guide for the current scoping review process [50]. 

### 2.2. Identify Research Questions

Research questions were developed by the research team based on their clinical experiences of providing intervention services to patients with TBI across the continuum of care. The research team anecdotally reported differences in outcome for many of their patients with TBI and questioned potential driving factors related to this. This led to two specific questions which guided this scoping review: (1) Which social determinants of health are most associated with TBI in adults? (2) What are the most common health disparities associated with TBI in adults?

### 2.3. Identify Relevant Studies

A research plan was developed to search three electronic databases—PubMed, MEDLINE, and CINAHL. PubMed and MEDLINE were selected for their wide ranging publications in the medicine and health fields and comprehensive reporting mechanisms. CINAHL was selected for its dedication to nursing and the allied health professionals that are such an integral part of intervention within the TBI population. Literature published between January 2015 and February 2023 was included in the search. This time frame was selected to provide the most updated information available. Furthermore, as practicing clinicians, the authors felt this time frame would most appropriately reflect the current clinical situations faced in daily practice. Search terms were identified that appropriately targeted the research questions and were grouped into three broad domains, traumatic brain injury, social determinants of health, and health disparities. A list of MeSH terms incorporated into the search, as well as potential synonyms and possible derivations of terms, are noted in Table 1. Importantly, the research team supplemented the computer generated databases search by hand searching the reference lists of appropriate full-text articles eventually identified through the review process. 

### 2.4. Inclusion and Exclusion Criteria

Determining appropriate inclusion and exclusion criteria was an iterative process and refined as study selection continued, in accordance with the previously established protocols [48]. Inclusion criteria consisted of: (1) Studies that addressed any aspect of SDoH and HDs within the adult TBI population; (2) peer reviewed, full-text published articles; (3) research that included meta-analysis, experimental and/or descriptive designs; (4) articles that broadly or specifically listed an individual’s SDoH features, or the HDs associated with TBI; and (5) published in English. Studies across the continuum of TBI, including the risk factors, acute, subacute, and/or long-term implications associated with TBI, were included in data analysis. Exclusion criteria consisted of review articles, commentaries, books, book chapters, dissertations, or conference abstracts. Papers with poor scientific rigor, and papers targeting only pediatric populations were eliminated. 

### 2.5. Study Selection Process

**Figure 1:**
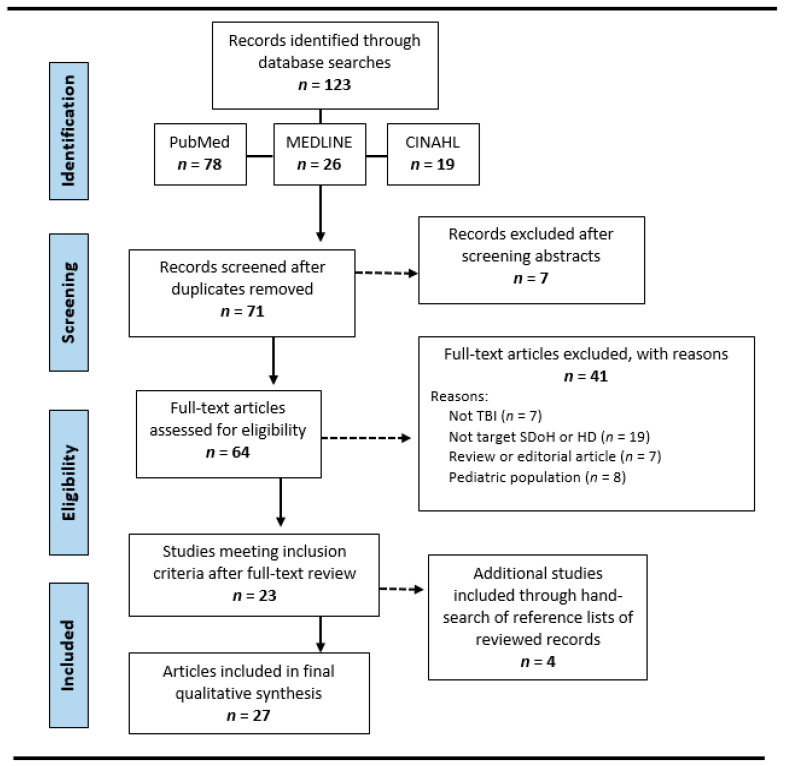
PRISMA diagram showing the process of identifying, screening, and determining eligibility for the scoping review.

The authors followed the PRISMA protocol for study selection as demonstrated in Figure 1. Searches of the three electronic databases yielded a total of 123 records. The research team eliminated duplicate records and then screened article titles and abstracts to verify inclusion criteria. Full-text screening occurred for the remaining 64 records. At this point, the research team conducted a secondary screening of the text because often the article title and abstract did not provide enough information to adequately establish exclusion criteria. This additional screening excluded another 41 studies, leaving a total of 23 articles (of the original 123) secured through the electronic databases search. The research team then procured another four full-text articles by hand-searching the reference sections of the selected articles. This resulted in a total of 27 articles included in the final analysis of this scoping review. 

The research team verified reliability of both the study selection and data extraction processes by following established guidelines using a peer-review process [51,52]. This review process began by two trained members of the research team independently screening all potential studies for title and abstract. A third reviewer was trained in the verification process and this team member randomly selected ten articles to review and independently verify those studies to ensure inclusion criteria were met. Next, the articles were screened for eligibility determination by two trained members of the research team who independently performed a full-text review of each article. Again, the third reviewer randomly selected ten articles to verify that they met the inclusion criteria. All 27 selected full-text articles were then reviewed by the authors to assess for adequate scientific rigor prior to initiating the data extraction stage. Over the course of the study selection verification process, reviewers agreed 96% of the time. When disagreements occurred, consensus was met by completing an additional review of the article, research team discussion, and resolution of discrepancies.

### 2.6. Data Extraction and Synthesis

The authors created a specific data collection chart to be used for each of the included studies that allowed for systematic data extraction. This chart’s points of data collection were revised as the review progressed based on ongoing discussion by the research team in an effort to ensure all applicable and relevant information was extracted. This is a typical method of the scoping review data collection process [47,48]. The final data collection chart consisted of 11 extraction points. Several points were dedicated to basic study information, such as authors, year of publication, research methodology used, and number of participants. Other data collection points were related to the type of TBI targeted, such as TBI severity level of the participant groups, or stage of TBI studied (i.e., acute, chronic). Additional data extraction points were used to evaluate each source based on the scoping review’s research questions related to SDoH and HDs.

Data extraction for each data point was recorded on a data summary sheet. This methodology is typical of the scoping review process as it allows for the tabulation of specific details and broad themes that may emerge related to the review of many articles. For example, this method allowed the research team to collect information from the SDoH data point in both large domains, such as race/ethnicity, SES, and language spoken; and also with more specific information related to these factors (i.e., specific race or ethnicity, urban or rural, primary and/or secondary language used). This was an important contribution of the scoping review process as it allowed this research team to fully explore the information presented in the articles related to their specific research questions. 

Again, a peer-review of accuracy occurred at the data collection stage, similar to that as conducted at the study inclusion stage. Both authors completed data extraction using the data summary sheet for all included articles. At this stage of the review process, reviewers agreed 90% of the time. Again, when disagreements were noted, discussion between the authors occurred, the article was reviewed again, and eventual consensus was reached. As discrepancies were resolved, the data collection sheet was updated.

The citations for the studies included in this analysis can be found in the Reference section [34,36,53,54,55,56,57,58,59,60,61,62,63,64,65,66,67,68].

## 3. Results

### 3.1. Description of Included Studies

Of the 27 studies included, the majority were published since 2020 (*n* = 17). Further breakdown of the included studies by 3-year increments indicated the following: 2023–2021 (*n* = 16), 2020–2018 (*n* = 6), and 2017–2015 (*n* = 5). Six countries were represented in the analysis, with the majority from the US (*n* = 21). Other global areas represented were Europe, (*n* = 2), Australia (*n* = 16), Canada (*n* = 1), Africa (*n* = 1), and Asia (*n* = 7). Fourteen studies incorporated all levels of TBI severity, two specifically targeted mild TBI, eight addressed moderate to severe TBI, and three looked only at severe TBI. Seven studies evaluated the impact of HD on TBI across the continuum of care, while the majority looked at TBI within the acute stage (*n* = 11). Six studies discussed HDs in the acute rehabilitation stage, and three additional studies looked at long-term HD associated with TBI. 

### 3.2. Social Determinants of Health Associated with TBI

**Figure 2:**
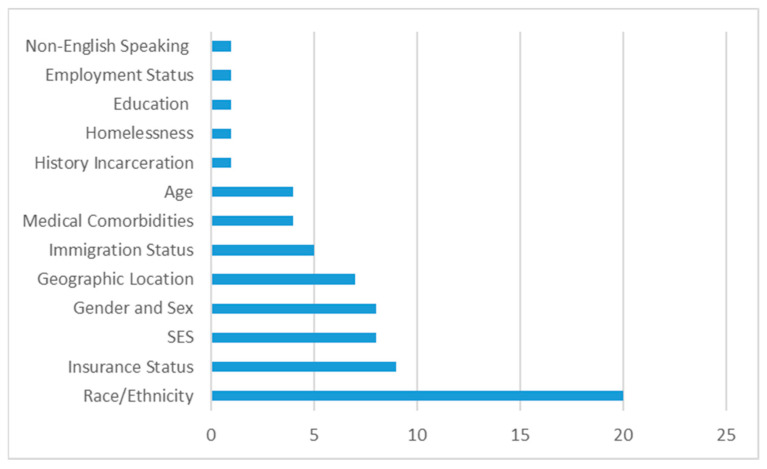
Frequencies of SDoH reported from review.

The first aim of this study was to determine which SDoH are most frequently associated with TBI in adults. From the data summary sheet, the authors recorded the frequency of any aspect of SDoH factors that were reported both within and across the included articles. This yielded a total of 70 data points, or instances, of SDoH factors reported within and across the studies. Analysis of these results indicated that the most frequent SDoH reported was race/ethnicity (*n* = 20). Races and ethnicities listed in the included studies were White, Black, Hispanic, non-White Hispanic, non-Hispanic White, Asian, and Indigenous. An individual’s insurance status was the next most commonly reported SDoH (*n* = 9), and generally reported as public versus private insurance. Socioeconomic status was next most frequently reported (*n* = 8), followed by gender and sex (*n* = 8). Access to care/geographic location of the individual was noted seven times. Immigration status (*n* = 5) was defined as native-born, immigrant, or undocumented immigrant. Multiple medical comorbidities (*n* = 4) was the next most frequently reported SDoH, and included both substance abuse and psychiatric illness. Age (*n* = 4) was another factor commonly reported. The remaining documented SDoH were each reported only once—history of incarceration, homelessness, education level, employment status, and language spoken. Figure 2 highlights the SDoH reported in the included articles. 

### 3.3. Noted Health Disparities 

The second aim of this study was to document the most common HDs associated with TBI in adults. The same methodology that was used to tabulate the number of SDoH was used to count the various HDs, yielding a total of 79 data points or instances of HDs reported within and across the articles. Analysis of the 27 included studies indicated that when the HDs were grouped according to SDoH domains, most of the HDs reported were due to race/ethnicity disparities (*n* = 50), followed by insurance status (*n* = 9). Health disparities were also commonly associated with sex/gender (*n* = 5), SES (*n* = 5), immigration status (*n* = 4), age (*n* = 3), and access to care/region/location (*n* = 3).

**Figure 3:**
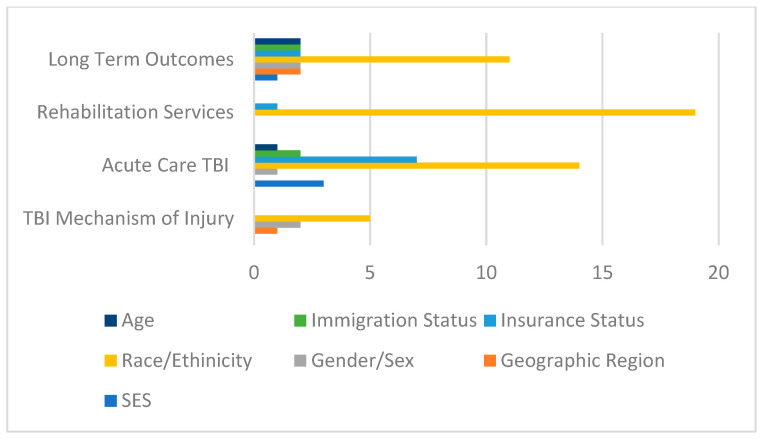
Health disparities reported in review grouped by SDoH and stage of TBI injury.

Health disparities were also analyzed according to the stage of TBI that the noted HD impacted. Eight HDs were noted in the risk and/or mechanism of injury category. Twenty-eight HDs were reported to occur within the acute stage category, and was the stage of TBI with the most reported HDs. Twenty HDs were noted to impact the rehabilitation stage of injury, while 22 HDs were reported within the long-term outcome stage. Figure 3 depicts each stage of TBI category and stratifies the HDs reported within each of those categories by SDoH.

Some HDs were reported multiple times within the studies reviewed. The most commonly noted HD was found in four reports and noted that Whites are more likely to be discharged to inpatient rehabilitation compared to Blacks or Hispanics. Another HD related to rehabilitation, and reported three times within the literature, was that Blacks were significantly more likely to be discharged home after acute inpatient rehabilitation. Several HDs were noted twice in the review, including (1) women are at a greater risk for TBI due to domestic violence, (2) racial/ethnic minorities are more likely to sustain a TBI from acts of violence, (3) racial/ethnic minorities experience longer wait times in the emergency department, (4) racial/ethnic minorities have an increased risk for inpatient mortality, (5) racial/ethnic minorities had longer hospital stays, (6) uninsured patients had fewer procedures during hospitalization, (7) individuals with public insurance had longer hospital stays compared to those with private insurance, (8) racial/ethnic minorities were less likely to receive follow-up services after hospitalization, and (9) racial/ethnic minorities had lower rates of referral to rehabilitation. A full listing of the recorded HDs can be found in Appendix A. 

## 4. Discussion

The purpose of this study was to systematically summarize the extensive and diverse scope of information available related to SDoH and HDs associated with TBI. Based on the results of this scoping review, the following section offers potential answers to the stated research questions, discusses ways to incorporate these results into clinical practice, and reveals areas of noted gaps in the literature that may prove to be beneficial for future research. 

### 4.1. Which Social Determinants of Health Are Most Associated with TBI in Adults?

Social determinants of health are the biological, social, and economic conditions in which people live and work that impact their overall health and well-being. These factors may be unmodifiable (i.e., race, age), or modifiable (i.e., education level, geographic location where an individual lives, employment status), even though those factors considered modifiable may be difficult to drastically change for some. Results from this scoping review indicated that race/ethnicity, a SDoH factor that is unmodifiable, was overwhelmingly the most commonly reported SDoH (*n* = 20) associated with TBI in adults. Race/ethnicity appeared to play a significant role in developing a risk for TBI, care received after TBI, and overall TBI-related health outcomes. 

The second most commonly reported SDoH was an individual’s insurance status, which may be considered a modifiable factor. Insurance status significantly impacts trauma outcomes for TBI patients. Insurance status can impact length of stay at the hospital, mortality, and treatment received. Importantly, insurance status may be largely dependent on other SDoH domains as well, such as SES, employment, or immigration status. Indeed, when results from this review group insurance status, SES, immigration status, and employment together into one SDoH category, this became the most commonly reported SDoH (*n* = 23). This larger category likely represents a variety of social and economic factors that are potentially being overlooked by some researchers and clinicians. There is a cycle regarding SES and it is difficult to change status in one generation. Indeed, it may take multiple generations to break the cycle, and involve multiple members of the family [69,70]. For example, a child from a lower SES may have difficulties reaching optimal academic achievement (i.e., limited funds to pay for school; employment trumps schooling given the need for insurance or income), which can subsequently affect the child’s adulthood and potential employability. 

Results from this indicated that the most frequently researched SDoH are related to race/ethnicity. While this is an extremely important consideration, researchers and clinicians should also be aware of other factors that are influencing the health of their patient, that do not seem to necessarily be studied as frequently. For example, no articles included in this review looked at health literacy or cultural considerations as a specific SDoH, and only a handful accounted for the language spoken of the individual, their housing situation, or educational level—all of which have been studied in other major health areas, but seemingly not as much the TBI population. These unaccounted SDoH in the TBI population may very well have real-life clinical implications. For example, the SDoH of social support may influence TBI recovery, as those patients with good social support may be better equipped to manage the emotional, cognitive, and physical consequences often associated with the long-term recovery of TBI; however, those patients without good social support may experience more significant psychological distress.

### 4.2. What Are the Most Common Health Disparities Associated with TBI in Adults?

The relationship between SDoH and HD in TBI is certainly complex and multifaceted. The results from this review demonstrated that biological, social, economic, and environmental SDoH contribute to health inequities related to TBI incidence, acute hospitalization, treatment options, and recovery. Health disparities most commonly reported were rooted in the race/ethnicity domain. Warren and Garcia found that TBI burden is higher in communities of color thus these people are disproportionately affected by TBI compared to non-Hispanic Whites [71]. These inequities likely stem from decades of structural racism and systematic oppression creating a disadvantage, in terms of health outcomes, for those who are racial/ethnic minorities [72,73]. Even when accounting for factors such as severity of TBI injury, and insurance status among patients of different races/ethnicities, research still indicates that there are significant differences in mortality and adverse outcomes [74,75,76]. Racial/ethnic disparities have been noted to influence risk for TBI, change how someone is treated in the emergency department (i.e., triaged lower, longer wait times, decreased rates of diagnostic procedures), and influence acute hospitalization (i.e., longer hospital stays, more likely to experience complications while hospitalized) [74,77,78]. Discharge rates to rehabilitation services in Black, Latinx, and Asian patients have been noted to be lower than for those who are White [79,80]. Odonkor et al. provide a comprehensive discussion of the impact of race during the rehabilitation stage and specifically noted that Black patients were less likely to be discharged to a higher level acute rehabilitation center [81]. Many studies have found that those of racial/ethnic minorities utilize hospital services less, and receive lower amounts of follow-up interventions than their White counterparts, which creates a health inequity that is ultimately linked to poorer health outcomes, including decreased rating of quality of life, decreased pleasure with family activities, and reduced chance of returning to work or school [71,75,82].

This review also noted several HDs related to insurance status. Differences can be seen in patients with insurance compared to those without it, but is also related to type of insurance (i.e., public versus private insurance) [75,83]. For example, length of hospital stay may be higher for TBI patients with Medicaid [75]. Lack of insurance has been associated with low procedural volume in patients with TBI [36,74,84]. Individuals who lacked insurance tended to have decreased use of in-hospital and post-hospital services, including inpatient, outpatient, and rehabilitation services [36,75,85]. Additionally, mortality rates have been reported to be higher in TBI patients with Medicare compared to those with private insurance [75].

Socioeconomic status was another contributing factor to HDs within the TBI population. Individuals in lower SES brackets and in lower income brackets have been reported to be at an increased risk of TBI [69]. Previous studies have shown a higher proportion of death due to injury among people with low SES [86,87]. From this review, HDs related to SES included changes related to likelihood of acute hospitalization admission and length of inpatient hospitalization [85,88]. Socioeconomic status has also been noted to impact an individual’s ability to receive follow-up care [89,90,91,92]. 

### 4.3. Study Strengths and Limitations

This review provides a comprehensive summary of the literature related to SDoH and HD impacting adults with TBI. A strength of this study is that it provides clinicians with a clear view of what social factors may be impacting their patient’s intervention after TBI. It also shows that there are a variety of SDoH that have been poorly documented or researched and the impact of these on an individual’s intervention after TBI has yet to be fully realized. Finally, this study synthesizes a variety of HDs noted throughout the continuum of TBI care, which can help clinicians evaluate potential risks for their patient at different stages of TBI recovery.

A limitation of this study was that only full-text articles attainable by the research team were included. This likely impacted which articles were able to be included in the review for data analysis and may have skewed results in favor of one SDoH or HD over another. Another limitation to this scoping review is the inherent heterogeneity of the TBI population that it evaluated, including etiology of TBI and associated individualized changes with the brain itself. This makes distillation of the research related to TBI difficult. The research team attempted to address this concern by creating a data collection chart that allowed for both broad and specific data analyses, which was able to capture the highly individualistic nature of the TBI population. Perhaps the greatest limitation faced by the research team was difficulties determining which SDoH most greatly influenced the resulting risk for HD. For example, the most commonly noted HD was that Whites are more likely to discharge to inpatient rehabilitation compared to Blacks or Hispanics. This may be influenced by a variety of SDoH in addition to race/ethnicity, such as insurance status, social support, employment, and health literacy; but given that most articles did not list each specific SDoH for each individual participant, it was difficult to verify the true factor, or likely factors, at the root of this HD. In situations such as this, the research team for this study categorized this into solely the main SDoH studied by the article to provide clarity and consistency for this review’s data collections. Given the inherent multifaceted and layered factors that impact one’s overall health, studying SDoH and the impact they have on an individual or group has proved to be difficult.

### 4.4. Future Directions

By reviewing the relationship between HDs and SDoH that contribute to TBI risk, researchers and clinicians can hope to develop targeted interventions to reduce TBI risk and improve outcomes. A review on the SDoH helps investigators to identify groups that are at an increased risk for sustaining a TBI, as well as those who are at an increased risk for not receiving timely care, not able to follow-up with treatment as recommended, or limited by healthcare seeking behaviors related to social or economic factors. By understanding these risk factors and identifying the gaps in care, policies and procedures can be developed that are more specifically aimed at reducing these risks or environmental hazards, and improving the overall access to healthcare. Better understanding of the relationship between an individual’s SDoH and their risk for HDs related to these, may also allow researchers and clinicians to develop targeted interventions to address the root causes of HD noted in the TBI population. For example, addressing health literacy, advocating to youth programs, creating improvements in clinical care, and advancing the use of telemedicine may all serve these at-risk populations and be ways to improve outcome after TBI [38]. Other ideas suggested that may reduce HDs include initiatives and policy change that focus on diversity, equity, anti-racism, and inclusion. This would also include anti-bias training for clinicians and researchers. Stereotypes and overt and covert biases influence clinical decision-making, therefore continued education on ways to increase awareness of how these biases impact clinical care and recommendations may be one strategy to decrease HDs. In addition to self-awareness, advocating for changed policy related to a variety of health care consideration is another tool important in reducing HDs [36]. Education and engagement with policy-makers related to the SDoH and HDs faced by people with TBI is an important step in reducing the health inequities found in this population [38]. Representation matters as well, so recruiting and training individuals from diverse groups that are reflective of the population they serve may also help to reduce HDs. Finally, further studies are needed to evaluate both SDoH and HDs, especially related to the potential predictive ability of a SDoH inventory to categorize risk for a possible health inequity. Additionally, other SDoH factors need to be explored by researchers within the TBI population, such as a person’s health literacy, their culture, their gender or sexual orientation, or housing situation.

## 5. Conclusions

Much yet remains to be discovered with regard to the relationship between SDoH and HDs within the TBI population. This review aims to provide a better understanding of the mechanisms underlying this relationship so that researchers and clinicians can identify at-risk populations, improve access to healthcare and rehabilitation services. Developing interventions that target these concerns can help to reduce the morbidity and mortality associated with TBI, and can encourage to work toward a future healthcare situation in which all individuals have similar experiences, steeped in quality and support. 

## Figures and Tables

**Table 1:** Key search terms for scoping review.

Domain	Key Words in the Title or Abstract	MeSH Terms
Brain Injury	brain OR injury OR “brain injury” OR “brain injuries” OR “traumatic brain injury”	brain injury OR traumatic brain injury OR TBI
Social Determinants of Health	“social determinants of health” OR “determinants of health” OR “health determinants”	social determinants of health OR SDOH OR health determinants OR healthcare determinants
Health Disparity	“health disparity” OR “health disparities” OR “health equity” OR ‘health inequity’	health dispar OR health equity

## Data Availability

The data that supports the findings of this study are available from the corresponding author upon reasonable request.

## Appendix A

Appendix A: Health disparities reported in review and grouped by stage of TBI injury.

### Appendix A.1. TBI Mechanism of Injury

Racial/ethnic minorities with an increased exposure to adverse childhood experiences have an increased risk of TBI [34].
Race does not inform concussion nondisclosure in collegiate athletes [66].
Racial/ethnic minorities are more likely to sustain TBI from acts of violence [34,61,76].
Racial/ethnic minorities more likely to sustain a TBI due to motor-pedestrian collisions [34].
People living in remote areas have an increased risk of neurotrauma following an accident [62].
Women are at a greater risk of TBI due to domestic violence [54,62].

### Appendix A.2. Acute TBI Care

Racial/ethnic minorities experience longer wait times in the emergency department [34,56].
Racial/ethnic minorities are more likely to leave the emergency department before seeing a physician [34].
Racial/ethnic minorities are more likely to receive testing for serum alcohol levels [85].
Racial/ethnic minorities are more likely to experience complications during inpatient hospitalization [74].
Racial disparity is associated with inpatient mortality risk [34,76].
Racial/ethnic minorities were less likely to die in the hospital [85].
Racial/ethnic minorities had longer hospital stays [81,85].
People of color experience more severe injuries [56].
Asians typically had the lowest injury severity at admission [82].
Racial differences exist between White patients and patients of color on self-rated injury severity scales that are discordant with severity as measured by more objective markers [56].
There were no statistically significant differences in discharge disposition comparing racial/ethnic groups [34,76,82].
SES disparity is associated with inpatient mortality risk [85].
SES disparity exists related to length of inpatient hospitalization [85].
People from lower SES areas had an increased chance of being hospitalized for intracranial injury [88].
Uninsured patients had fewer procedures during hospitalization [74,84,85].
Publicly insured patients have higher odds of undergoing surgical management for traumatic SDH compared to self-pay patients [63].
A higher percentage of self-pay TBI patients die in the hospital [63].
Medicare patients are less likely to undergo craniectomy [63].
Individuals with public insurance had longer hospital stays compared to those with private insurance [84,85].
There is no association between in-hospital mortality rate and immigrant documentation status [68].
Undocumented immigrants had a longer average hospital length of stay [68].
Older patients are less likely to undergo craniectomy [75].
Females are less likely to undergo craniectomy [75].
Women receive different inpatient care depending on age and severity of TBI [60].

### Appendix A.3. TBI Rehabilitation Services

Whites more likely to discharge to inpatient rehabilitation than Blacks or Hispanics [55,74,81,82].
Compared to Whites, Blacks were significantly more likely to be discharged home [36,71,81].
Racial/ethnic minorities are less likely to receive follow-up services after hospitalization [36,81].
Racial/ethnic minorities have lower rates of referral to rehabilitation [55,82].
Racial/ethnic minorities are more likely to receive fewer rehabilitation minutes [34].
Racial/ethnic minorities have a higher likelihood of early discharge from rehabilitation [34].
Hispanic patients with comparable injury severity and insurance status receive different discharge dispositions post-TBI, even in regions in which Hispanics are the demographic majority [55].
White patients are admitted to acute rehabilitation significantly faster than people of color [56].
Hispanic ethnicity was associated with a greater odd of discharge to home [71].
People of color had lower admission and discharge FIM scores [71].
Asian patients at Trauma Level 1 hospitals were more likely to be discharged to acute rehabilitation if they had private versus public insurance [57].
Uninsured patients were the least likely to go to inpatient rehabilitation [74,81].

### Appendix A.4. Long-Term TBI Outcomes

Whites more likely to receive palliative care encounters than Black and Hispanic patients [67].
Whites more likely to refuse PEG placement after a palliative care consult [67].
After controlling for covariates, Blacks had a greater rate of depression than Whites, and similar rate as Hispanics [81].
Blacks had lower life satisfaction rates over time compared to Whites [81].
Racial/ethnic minorities more likely to report higher levels of caregiver burden after TBI [81].
Blacks were more likely to report more challenges coping with depression [81].
Racial/ethnic minorities more likely to experience long-term adverse outcomes [34].
Black patients with private insurance and Black patients with public insurance had poorer outcomes than privately insured White patients [75].
Hispanics with TBI showed greater participation than non-Hispanic Whites in the area of being out and about in the community 1-year post-injury [64].
Asians failed to make similar improvements as noted in Hispanics and Whites between rehabilitation discharge and 1 year post-TBI follow-up [82].
Race was not a significant predictor of outcome or mortality [71].
Sex was not a significant predictor of outcome or mortality [61].
There are gender differences related to self-reported post-concussion syndrome symptoms, which are likely based in anxiety sensitivity [53].
Older patients are more likely to have poorer outcomes [81].
Age was not a significant predictor of outcome or mortality [61].
SES was not a significant predictor of outcome or mortality [61].
Nativity, moderated by the residential proportion of foreign language speakers, predicted productive activity 1-year post TBI [59].
Indigenous populations of Canada faced inadequate resources, social problems, and challenges within the health care system [62].
Lack of health insurance was significantly associated with decreased use of post-hospital healthcare services [36].
Medicare and Medicaid patients of all race categories had poorer outcomes than privately insured White patients [81].
People with TBI living in more rural areas traveled significantly further to access post-discharge health services and had decreased amounts of follow-up healthcare appointments [58].
Countries with a high incidence of TBI has disproportionately higher research publications compared to countries with lower TBI populations [65].
